# Fiber post cemented using different adhesive strategies to root canal dentin obturated with calcium silicate-based sealer

**DOI:** 10.1186/s12903-024-04963-7

**Published:** 2024-10-25

**Authors:** Lalita Patthanawijit, Kallaya Yanpiset, Pipop Saikaew, Jeeraphat Jantarat

**Affiliations:** Department of Operative Dentistry and Endodontics, Faculty of Dentistry, Mahidol University, 6 Yothi street Ratchathewi, Bangkok, 10400 Thailand

**Keywords:** Calcium silicate-based sealer, Bioceramic sealer, Fiber post, Bond strength, Universal adhesive

## Abstract

**Background:**

Calcium silicate-based sealer has favorable properties for root canal filling, including hydroxyapatite formation during the setting process. However, this process can cause difficulty during post space preparation when the sealer is set. Additionally, the remaining sealer could interfere with the bond strength of fiber post to root canal dentin. The different adhesive strategies and fiber post cementation time may affect the bond strength of the fiber post. Thus, the objective of this study was to evaluate the effect of etching modes of Scotchbond™ Universal Plus adhesive and post cementation time on the push-out bond strength of a fiber post cemented in root canals obturated with calcium silicate-based sealer.

**Methods:**

Fifty-four teeth were randomly allocated to 6 groups (*n* = 9) based on etching modes: self-etch (SE) or etch-and-rinse (ER); post space preparation and cementation time: immediate (Im) or 7-day delayed (De): Im-Im, Im-De, and De-De. The root canals were obturated with calcium silicate-based sealer and the post space preparation was performed. The fiber post was cemented using RelyX™ Universal resin cement according to each group’s design. For the push-out bond strength test, 1-mm slices of the coronal, middle, and apical regions were tested using a universal testing machine. The failure mode analysis was determined using a stereomicroscope. The data was analyzed with three-way analysis of variance.

**Results:**

No negative effects of etching modes, post space preparation or cementation time on push-out bond strength were detected (*p* > 0.05). Additionally, the root canal region also did not significantly affect the bond strength (*p* > 0.05).

**Conclusion:**

No significant differences were observed between the etching modes, post space preparation and cementation time and among root canal regions.

**Clinical relevance:**

The different etching modes of adhesive and post cementation time did not affect the bond strength of fiber post in calcium silicate filled-root canal.

## Background

Endodontically treated teeth are commonly associated with extensive loss of coronal tooth structure [1] and may need a post and core prior to the definitive restoration [2]. Fiber posts have a similar modulus of elasticity to dentin and provide better esthetic outcomes than metal cast posts. They can distribute stress to the root canal dentin and tend to debond before causing root fracture [3].

Calcium silicate-based sealers (CSS) have become an alternative for root canal obturation due to their excellent flowability and biocompatibility. The main compositions are di-calcium silicate, tri-calcium silicate, calcium phosphate, colloidal silica, and calcium hydroxide, which can form a tag-liked structure at the calcium silicate/dentin interface [4]. According to these properties, CSS can flow into the irregularities of the root canal and provide sealing ability. Thus, these sealers have become popular and widely used. The CSS showed higher solubility than epoxy resin-based sealer [5]. Additionally, the fresh CSS are generally more soluble than its set state. In contrast, set CSS is more difficult to remove from the root canal due to the hardness of set CSS [6]. In addition, the remaining CSS can interfere with the bonding efficiency of resin cements resulting in the lower bond strength of fiber-reinforced posts [7–11]. Thus, immediate post space preparation may be easier to remove the CSS in the post space.

Pretreatment of the post space and the post cementation time have been reported as important factors related to the bond strength of the post [12]. Nonetheless, some studies showed the cementation time has no influence on the bond strength of the post in canal obturated with calcium silicate-based sealers [13, 14]. while a recent study reported immediate post cementation after root canal obturation with calcium silicate-based sealer showed higher bond strength [15]. Thus, the effect of the post cementation time remains controversial.

The resin cement system also affects the bond strength of the fiber post [12]. There are 3 types of resin cement according to etching modes: – etch-and-rinse (ER), self-etch (SE), and self-adhesive resin cement. Although the self-adhesive resin cement is easier to use and less time consuming, self-adhesive system showed lower fiber post bond strength compared with conventional adhesive system [16]. A systematic review and meta-analysis study concluded that using resin cement with ER mode is more favored than SE system when bonded to calcium silicate-based cement. The reasons are acid-etch technique can enhance the wettability of material and increase the surface’s porosity resulting in micro-retention zone during adhesion [17]. Recently, adhesive materials have been developed for better dentin-material bonding efficiency. Universal adhesives were recently introduced to the market and permit the use of ER or SE strategies. However, there is no evidence to support the efficiency of each etching mode on the root dentin of CSS-filled root canals. Thus, the aim of this study was to analyze the effect of adhesive etching modes and cementation time on post push-out bond strength (POBS). The null hypotheses were (1) There was no difference between etch-and-rinse and self-etch mode adhesive on the push-out bond strength of the post cementation. (2) There was no difference between immediate and delay post cementation time on the push-out bond strength of the post. (3) There was no different push-out bond strength of the post on each root canal area. (4) There was no different interaction between the post cementation time, etching mode, and root canal area.

## Materials and methods

### Tooth selection

Sixty human mandibular premolars which extracted for orthodontic reasons were collected from 14-to-45-year-old patients and stored at room temperature in a 0.1% thymol solution (M Dent, Bangkok, Thailand) and used within six months. Sound teeth with complete root formation and 15 ± 1 mm root length were selected. The samples were examined radiographically in the mesiodistal and buccolingual directions to confirm a single root canal, the degree of curvature not greater than 5º, and the buccolingual to mesiodistal ratio not greater than 1.5 at 5 and 10 mm.

### Sample preparation

The teeth were cut perpendicular to their longitudinal axis using a low speed cutting machine (IsoMet™ Low Speed Saw; Buehler Ltd., IL, USA) with constant water lubrication to standardize the sample length at 16 mm. The working length was set 1 mm from the apex. The root canals were prepared with a 55 and taper 0.04 rotary file (M3L Platinum; UNITED DENTAL GROUP, Changzhou, China) and irrigated with 2.5% NaOCl (M Dent, Bangkok, Thailand) during preparation. The final flush used 3 ml 17% ethylenediaminetetraacetic acid (M Dent, Bangkok, Thailand) for 1 min, then 5 ml 2.5% NaOCl for 1 min.

Fifty-four teeth randomly selected teeth were obturated with a gutta-percha cone (Spident Ltd., Incheon, Korea) and CSS using the sealer-based technique. The specimens were randomly assigned into 6 groups (*n* = 9) according to the etching modes of Scotchbond^™^ Universal Plus adhesive (SUP), post space preparation and cementation time (Immediate (Im) or Delayed (De)): G1:SE/Im-Im, G2:ER/Im-Im, G3:SE/Im-De, G4:ER/Im-De, G5:SE/De-De, and G6:ER/De-De (Table 1). The G5 and G6 specimens were covered with a temporary filling (Caviton, GC Asahi corp., Aichi, Japan) and kept in a humid atmosphere at 37ºC for 7 days before post space preparation.

**Table 1:** The sample groups were divided according to etching modes and time

Groups	*n*	Sealer	Etching modes	Post space preparation	Cementation time
G1: SE/Im-Im	9	iRoot SP	Self-etch	Immediate	Immediate
G2: ER/Im-Im	9	iRoot SP	Etch-and-rinse	Immediate	Immediate
G3: SE/Im-De	9	iRoot SP	Self-etch	Immediate	Delayed 7 days
G4: ER/Im-De	9	iRoot SP	Etch-and-rinse	Immediate	Delayed 7 days
G5: SE/De-De	9	iRoot SP	Self-etch	Delayed 7 days	Delayed 7 days
G6: ER/De-De	9	iRoot SP	Etch-and-rinse	Delayed 7 days	Delayed 7 days
Control SE	3	No	Self-etch	Immediate	-
Control ER	3	No	Etch-and-rinse	Immediate	-

The remaining 6 teeth were obturated using the thermoplasticized gutta-percha injection technique with no sealer and randomly assigned into 2 groups according to etching modes and served as controls.

### Post space preparation and post cementation

The G1-G4 specimens were prepared immediately after root canal obturation. The gutta-percha was removed using heat instrument leaving 4 mm of apical sealing, then drilled with a finishing drill. The post spaces were irrigated with 5 ml of distilled water, then dried with paper points. The G3 and G4 specimens were covered with Caviton and kept in a humid atmosphere at 37ºC for 7 days before post cementation.

For the delayed post space preparation groups, the Caviton was removed with an ultrasonic tip, and the post spaces was prepared using the same process as the immediate post space preparation.

The fiber posts were scrubbed with cotton pallet soaked with 70% ethyl alcohol and gently air dried. SUP adhesive was then applied using microbrush for 20 s and gently air dried for 5 s. The adhesive was applied in the post spaces following application of each mode in Table 2. The RelyX^™^ Universal resin cement was deposited, then the fiber post was seated to full depth using finger pressure and photo-activation was performed for 20 s using a light-curing unit (Elipar™ DeepCure-S LED Curing Light; 3 M ESPE, Australia) with light intensity of 1470 mW/cm^2^. The samples were immersed in distilled water and kept in a humid atmosphere at 37ºC for 7 days for completed setting.

**Table 2:** The sample groups were divided according to etching modes and time

Materials	Composition	Application		Lot number
iRoot SP (Innovation BioCeramix Inc., BC, Canada)	Tri-calcium silicate, di-calcium silicate, calcium phosphate monobasic, zirconium oxide, tantalum oxide, calcium hydroxide, and thickening agent	Dry the canal using paper points. Remove the syringe cap from the iRoot SP syringe and securely attach a disposable Intra Canal tip to the hub of the syringe. Insert the tip of the syringe into the canal no deeper than the coronal one-third (1/3). Gently and smoothly dispense a small amount (1–2 reference markings) of iRoot SP into the root canal by compressing the plunger of the syringe. Using a #15 hand file or any comparable hand file, lightly coat the canal walls with the existing iRoot SP in the canal. Then coat the master gutta percha cone with a thin layer of iRoot SP and very slowly insert it into the canal. The master gutta percha cone will carry sufficient iRoot SP to the apex.		21003SP
Scotchbond^™^ Universal Plus adhesive (3 M ESPE, MN, USA)	MDP Phosphate monomer, HEMA, Vitrebond^™^ Copolymer, Filler, Ethanol, Water, Initiators, mixture of silanes, dual-cure accelerator, Dimethacrylate resins contain a BPA derivative-free	**Etch-and-rinse mode.** Apply a phosphoric acid gel (37%) in the root canal for 15 s. Rinse the entire canal with distilled water for 30 s and dry with paper points. Then, actively apply SUP using a microbrush for 20 s and then dry with paper points.	**Self-etch mode**. Actively apply SUP using a microbrush for 20 s and then dry with paper points.	8,321,974
RelyX^™^ Universal resin cement ((3 M ESPE, MN, USA)	BPA derivative-free dimethacrylate monomers, Phosphorylated dimethacrylate adhesion monomers, Photoinitiator system, Novel amphiphilic redox initiator system, Radiopaque fillers and rheological additives, Pigments	Insert the micro mixing tip to the syringe by putting the triangle key of the tip point to the white arrow on the syringe. Rotate the tip 90 degree clockwise until stop to unlock the syringe followed by inserting the elongation tip to the micro mixing tip until it locks. Apply moderate centric force without bending the plunger. Discard a small amount of the cement and then insert the tip into the root canal and inject the cement. After completion, remove the micro mixing tip by rotating it anticlockwise and pull the tip out. The cement syringe is self-sealing, store the syringe without a mixing tip.		8,493,604
DT Light-Post Illusion X-RO #1 (RTD, St. Egreve, France)	Radiopaque quartz fibers, Epoxy resin matrix, Pigment	Use a finishing drill that matches with the post size for enlarging the root canal. Rinse the root canal using distilled water to remove the dentin chips, then dry the root canal with paper points. Check the post fit in the root canal.		533,452,202

### POBS test

**Fig. 1:**
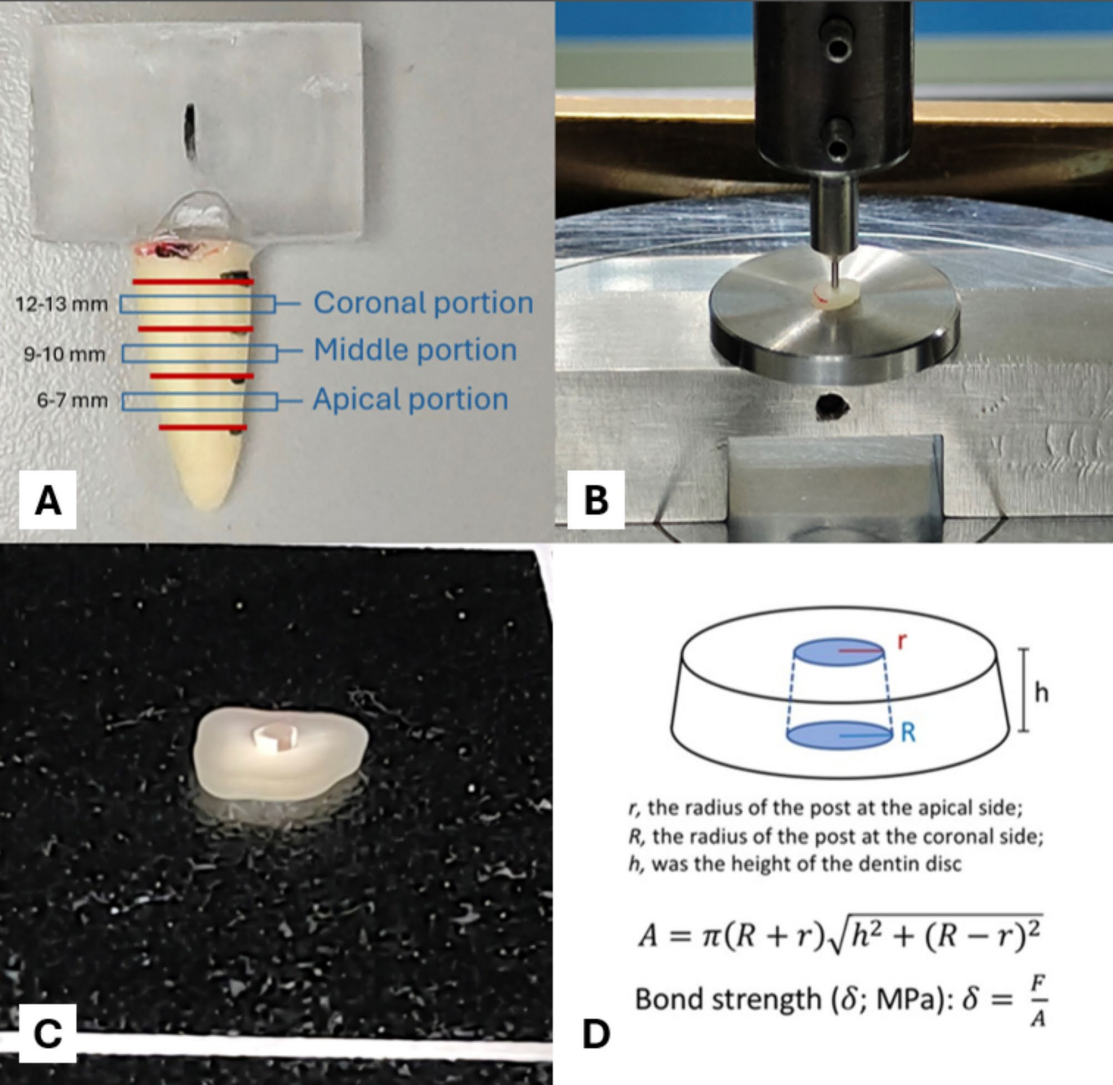
(**A**) The root slices preparation. (**B**) Push-out bond strength test: the root slice was placed apical-side up, and the plugger head was located at the center of the fiber post. (**C**) The root slice after the push-out bond strength test. (**D**) The area of bonded surface equation and bond strength equation

After 7 days, each specimen was sliced into three 1-millimeter-thick slices using IsoMet^™^ with constant water irrigation to represent the fiber post coronal, middle, and apical portion (Fig. 1A). The thickness of each slice was determined using a digital vernier caliper (ABSOLUTE Digimatic Caliper Series 500; Mitutoyo Corp., Kanagawa, Japan) within 0.1 mm.

Each slice was attached to a universal testing machine (Intron 5566; Intron Ltd., Buckinghamshire, UK) with the apical side facing upward (Fig. 1B). The post segment was loaded with a cylindrical plunger centered on the post segment. A 1 mm/min crosshead speed was applied until the post was completely dislodged (Fig. 1C). The maximum force at the point of extrusion of the post segment was recorded as *F* in Newton (N). The bonded area (A) of the post and the bond strength $\:\left(\delta\:\right)$ in Mega Pascal (MPa) were calculated using the equations (Fig. 1D).

### Failure mode analysis

Finally, the slices were observed using a stereomicroscope at 50X magnification to identify the failure type. Failure was quantified as a percentage and categorized into six categories [15]: Adhesive failure at the cement/dentin interface, post/cement interface, cohesive failure within the resin cement, within post, within dentin, and mixed failure.

The specimens were examined by a single operator, who determined that more than 75% of failures fell into a category. Mixed failure was determined if more than one mode of failure was demonstrated, and each category did not exceed 75%.

### Statistical analysis

Statistical analyses were performed using SPSS version 26.0.0.0 (SPSS Inc., Chicago, IL). Descriptive statistics were used to describe the findings and failure modes. The post space preparation and cementation time were used as one factor. A three-way mixed analysis of variance (ANOVA) was performed to evaluate the effect of time of etching mode, post space preparation and post-cementation, and root canal area. Pairwise comparisons were performed using the Bonferroni test. Statistical significance was defined at *P* < 0.05.

## Results

The mean POBS of the study groups are reported in Table 3. Three-way analysis of variance indicated that there was no difference between adhesive etching modes, fiber post cementation time, and root area (*P* > 0.05) and there was no interaction between the three factors (*P* > 0.05).

**Table 3:** Push-out bond strength of the fiber post (MPa) and failure modes of each group

Group	Each root area		All root area	Failure modes (Sample number (percent))					
	**Root area**	**MPa** **(Mean ± SD)**	**MPa** **(Mean ± SD)**	**Adhesive**		**Cohesive**			**Mixed**
				**ce-dt**	**ce-po**	**ce**	**po**	**dt**	
G1 SE/Im-Im	Coronal	14.09^a^ ± 2.88	12.77 ± 3.61	2 (22%)	4 (45%)	0	0	0	3 (33%)
	Middle	10.97 ^a^ ± 5.50		5 (56%)	2 (22%)	0	0	0	2 (22%)
	Apical	13.25 ^a^ ± 4.30		9 (100%)	0	0	0	0	0
G2 ER/Im-Im	Coronal	11.38 ^a^ ± 3.10	13.06 ± 3.04	2 (22%)	5 (56%)	0	0	0	2 (22%)
	Middle	13.37 ^a^ ± 4.04		3 (33%)	1 (11%)	0	0	0	5 (56%)
	Apical	14.43 ^a^ ± 4.88		2 (22%)	5 (56%)	0	0	0	2 (22%)
G3 SE/Im-De	Coronal	16.32 ^a^ ± 4.27	15.16 ± 2.78	0	7 (78%)	0	0	0	2 (22%)
	Middle	14.60 ^a^ ± 4.09		1 (11%)	4 (45%)	0	0	0	4 (45%)
	Apical	14.57 ^a^ ± 2.57		4 (45%)	4 (45%)	0	0	0	1 (11%)
G4 ER/Im-De	Coronal	14.52 ^a^ ± 3.77	15.50 ± 2.65	0	5 (56%)	0	1 (11%)	0	3 (33%)
	Middle	16.59 ^a^ ± 4.18		1 (11%)	5 (56%)	0	0	0	3 (33%)
	Apical	15.39 ^a^ ± 4.12		0	8 (89%)	0	0	0	1 (11%)
G5 SE/De-De	Coronal	12.12 ^a^ ± 5.30	11.79 ± 3.89	2 (22%)	1 (11%)	0	0	0	6 (67%)
	Middle	10.51 ^a^ ± 4.70		4 (45%)	2 (22%)	0	0	0	3 (33%)
	Apical	12.75 ^a^ ± 3.87		3 (33%)	1 (11%)	0	0	0	5 (56%)
G6 ER/De-De	Coronal	12.97 ^a^ ± 3.57	13.26 ± 2.30	1 (11%)	2 (22%)	0	0	0	6 (67%)
	Middle	14.28 ^a^ ± 4.23		0	5 (56%)	0	0	0	4 (45%)
	Apical	12.54 ^a^ ± 2.36		0	3 (33%)	0	0	0	6 (67%)
Control SE	Coronal	17.16 ^a^ ± 2.94	15.89 ± 2.12	0	3 (100%)	0	0	0	0
	Middle	15.96 ^a^ ± 2.64		0	2 (67%)	0	0	0	1 (33%)
	Apical	14.56 ^a^ ± 1.81		0	1 (33%)	0	0	0	2 (67%)
Control ER	Coronal	13.18 ^a^ ± 2.13	11.91 ± 1.45	0	2 (67%)	0	0	0	1 (33%)
	Middle	10.85 ^a^ ± 5.59		1 (33%)	0	0	0	0	2 (67%)
	Apical	11.71 ^a^ ± 0.22		1 (33%)	1 (33%)	0	0	0	1 (33%)

SE, self-etch; ER, etch-and-rinse; Im-Im, immediate post space preparation and immediate post cementation; Im-De, immediate post space preparation and delayed post cementation; De-De, delayed post space preparation and delayed post cementation; ce, cement; dt, dentin; po, post

Mean and SD value of the push out bond strength in MPa. Mean value with the different letter was significantly different at *P* < 0.05

**Fig. 2:**
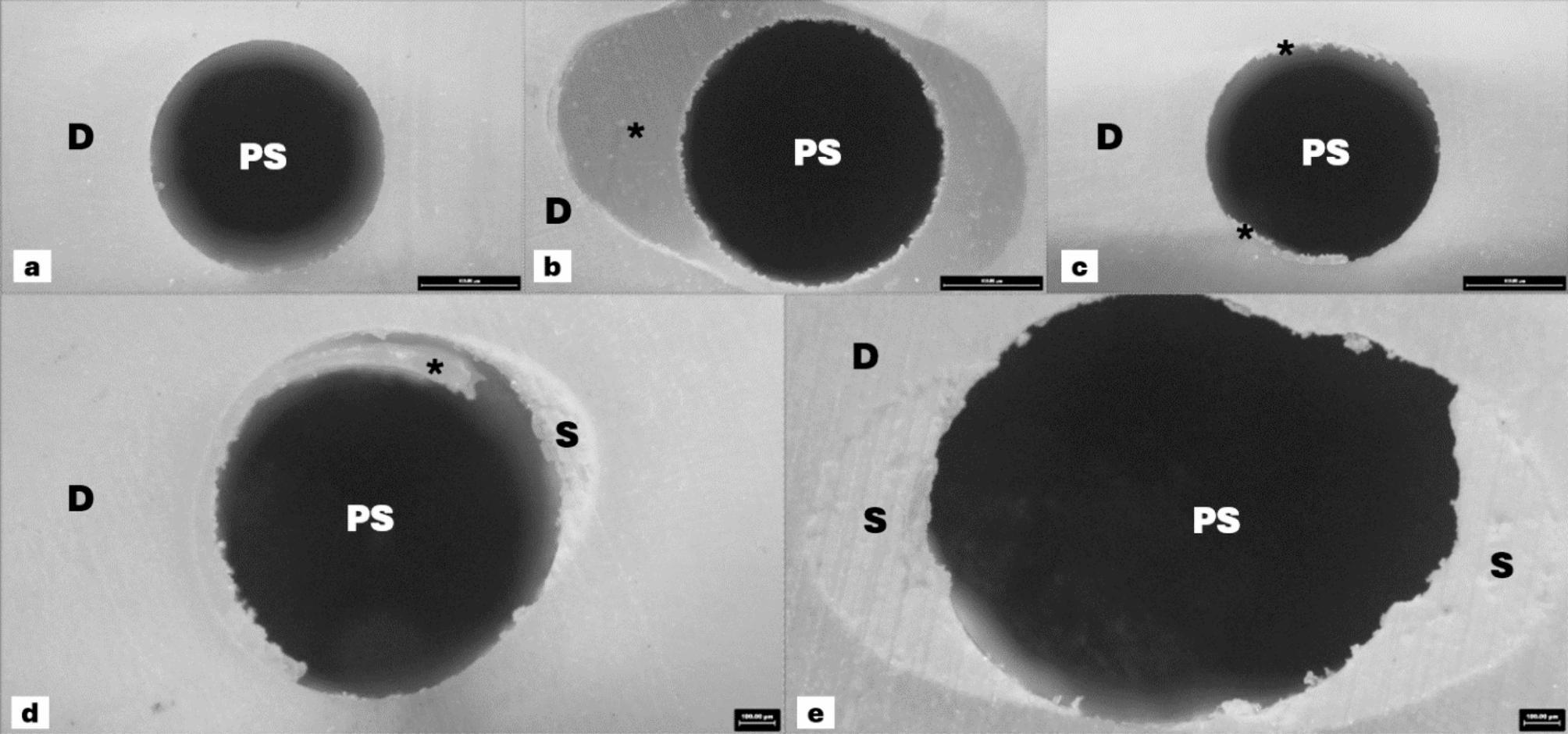
Representative stereomicroscope images of the failure modes. (**a**) Adhesive failure between the cement-dentin interface. (**b**) Adhesive failure between the cement-post interface. (**c**-**e**) Mixed failure. D, dentin; PS, post space; S, calcium silicate-based sealer; *, resin cement

From stereomicroscope analysis, the representative image of failure mode was demonstrated in Fig. 2. The failure mode analysis revealed that adhesive failure between the cement and post interface was the most prevalent failure mode, followed by mixed failure (Table 3).

## Discussion

Our results indicated that there was no significant difference in etching modes, post space preparation, or root area on the POBS of the fiber post (*p* > 0.05). Therefore, all anticipated null hypotheses were not rejected. Regarding the etching modes, our findings are consistent with Velho et al. [18] who found that no significant difference in bond strength between ER or SE of Single Bond Universal adhesive. In contrast, some studies demonstrated that using SE mode generated higher POBS [19, 20] due to the lower technique sensitivity, whereas the lower POBS in ER mode was reported to be due to incomplete resin infiltration into the etched dentin [21]. However, it was reported that CSS- contaminated substrates might interfere with bonding efficiency [13]. Therefore, this might be the reason for the similar POBS of SUP when used with different etching modes.

Calcium silicate-based sealers are the new generation of endodontic sealers which show outstanding biocompatibility, and bioactivity. These sealers can effectively adhere to intra-radicular dentin and have been reported superior bond strength compared to alternative sealers [22]. The hydration reaction between the sealer and dentinal fluids leads to the formation of tag-like structures within the dentin. In addition, this sealer can be used as a main component of the root canal filling with less gutta percha because of slightly expansion after setting [23]. Consequently, the complete setting of bioceramic sealers pose challenges during removal and results in a greater amount of residual sealer presence compared to epoxy resin-based sealers, particularly in more oval-shaped canals [11].

In our study, the experimental groups represented different clinical situations. The De-De situation is commonly encountered in clinical practice. After completing root canal treatment, the patient needs an additional visit for post space preparation and cementation, where the sealer is completely set. The set CSS, which is mainly composed of tri- and di-calcium silicate, acts as a physical barrier between the adhesive and dentin. This is consistent with our failure mode analysis of the De-De groups showing a high percentage of mixed failure, regardless of etching modes (Table 3). In addition, a study reported the difficulty of CSS removal for conventional retreatment due to the hardness of the set CSS [6], as the reported hardness of the completely set CSS was high [4]. Though the De-De groups’ POBS were not significantly different from the other groups, the difficulty of post space preparation due to the hardness of set CSS and the possibility of complication, such as deviation or perforation, is concerned.

The Im-Im groups represent cases in which the fiber post is cemented during the same visit as the root canal obturation, while the sealer was not completely set [24–26]. It was expected that the Im-Im groups would demonstrate a higher POBS due to the early removal of the unset sealer. However, the POBS was not significantly different from the other groups. This is likely because the complete removal of the unset sealer cannot be achieved [10, 13]. Furthermore, the alkaline pH of calcium hydroxide by-products during the setting reaction might affect the acidic pH bonding agent of the resin cement [27]. Moreover, during the hydration and precipitation reaction of CSS, the final products are calcium hydroxyapatite (Ca_10_(PO_4_)_6_(OH)_2_) and water (H_2_O) [4]. The water generated from this reaction may interfere with the polymerization reaction of resin cement which is hydrophobic.

**Fig. 3:**
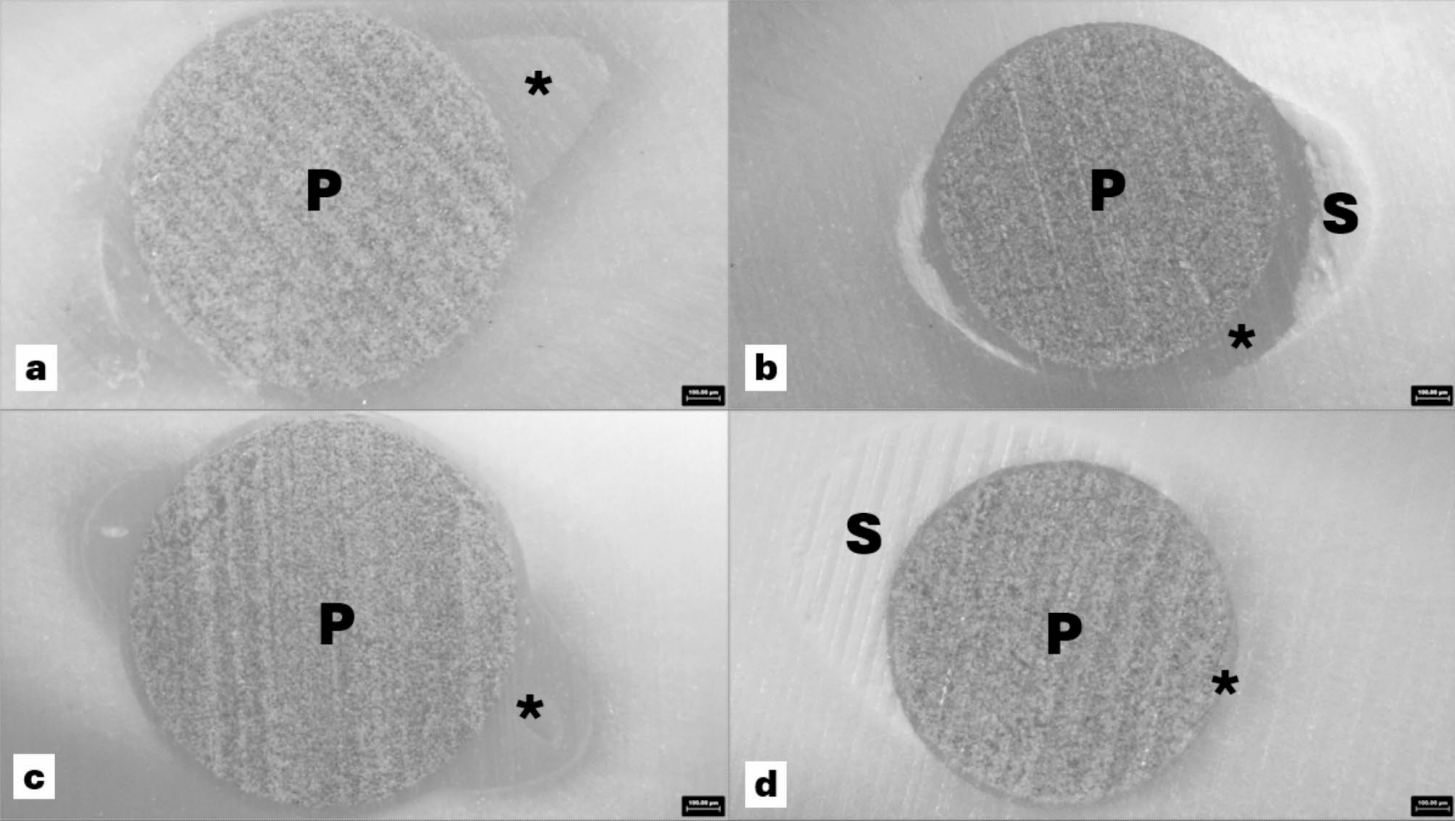
Representative stereomicroscope images of sample slices of the Im-De groups (**a**, **c**) compared with the De-De groups (**b**, **d**). P, fiber post; *, resin cement; S, calcium silicate-based sealer

The Im-De groups represent the situation where the post space preparation is immediately performed following root canal obturation and requires an additional visit for post cementation. During this procedure, the unset sealer that coated the dentinal wall was rinsed off in a similar manner as in the Im-Im groups to eliminate the sealer interface. The remaining sealer is left for complete setting before fiber post cementation. Our findings revealed that the POBS in the Im-De groups were higher compared with the other two techniques regardless of etching modes. However, no significant effect was detected (*p* > 0.05). As previously mentioned, the substrates were contaminated with CSS, which may act as a physical barrier between dentin-adhesive interfaces. From the experimental observations, the Im-De groups had less CSS remaining on the dentinal wall compared to the De-De groups (Fig. 3). There was less root canal dentin wall available for the bonding surface in the De-De groups due to the relatively weak acidicity of SUP (pH ≈ 2.7) [28], the etching effect of SUP might be too weak to eliminate the residual sealer layer.

This investigation revealed no significant differences in the timing of post space preparation and cementation within the root canal area (*p* > 0.05). However, other studies reported higher bond strength in the coronal region compared with other regions [29, 30]. This difference may be attributed to the larger size and greater number of dentinal tubules in the coronal part, facilitating enhanced access and adhesive application [19]. Additionally, the higher intensity of the curing light in the coronal area may also contribute to this effect. Importantly, these studies used an epoxy resin-based sealer and gutta-percha for root canal filling, which had shown no negative influence on the bond strength of fiber posts [14, 31]. In the present study, the effect of the root canal regions on the POBS was not significant. These results might be explained by the presence of the remaining CSS, particularly in the coronal portion, which have oval-shaped root canals (Fig. 3). In this study, the mandibular human premolar was selected because of its lower anatomical complexity and high availability. However, the general anatomy of the lower premolar is rounder at the apical and more oval at the coronal level of the root, thus the variety of oval shape can affect the resin cement thickness and the bond strength of the fiber post. A cement thickness more than 0.35 mm significantly reduces the bond strength of the post [32]. Therefore, the bucco-lingual to mesio-distal width ratio of the root canal was measured to control that the root canal shape of specimens were as round as possible. However, the coronal portion of the root canal is always oval-shaped and cannot be controlled.

## Conclusion

Within the limitations of this study, there was no effect of etching modes, post space preparation and cementation time, and root area on post space preparation. However, the difficulty of post space preparation in the De-De groups is concerned. Therefore, dentists need to pay more attention when preparing the delayed post space preparation in clinical practice. The immediate post space preparation may be a good choice to avoid clinical errors.

## Data Availability

No datasets were generated or analysed during the current study.

## Abbreviations

SUP: Scotchbond™ Universal Plus adhesive
POBS: Push-out bond strength
CSS: Calcium silicate-based sealer
SE: Self-etch
ER: Etch-and-rinse
Im: Immediate
De: Delayed
NaOCl: Sodium hypochlorite
